# Modelling reassurances of clinicians with hidden Markov models

**DOI:** 10.1186/s12874-018-0629-0

**Published:** 2019-01-09

**Authors:** Valentin Popov, Alesha Ellis-Robinson, Gerald Humphris

**Affiliations:** 1 School of Mathematics and Statistics, University of St Andrews, The Observatory, Buchanan Gardens, St Andrews, KY16 9LZ UK; 2 School of Medicine, University of St Andrews, North Haugh, St Andrews, KY16 9TF UK

**Keywords:** Reassurance, Hidden Markov models, Fixed effects

## Abstract

**Background:**

A key element in the interaction between clinicians and patients with cancer is reassurance giving. Learning about the stochastic nature of reassurances as well as making inferential statements about the influence of covariates such as patient response and time spent on previous reassurances are of particular importance.

**Methods:**

We fit Hidden Markov Models (HMMs) to reassurance type from multiple time series of clinicians’ reassurances, decoded from audio files of review consultations between patients with breast cancer and their therapeutic radiographer. Assuming a latent state process driving the observations process, HMMs naturally accommodate serial dependence in the data. Extensions to the baseline model such as including covariates as well as allowing for fixed effects for the different clinicians are straightforward to implement.

**Results:**

We found that clinicians undergo different states, in which they are more or less inclined to provide a particular type of reassurance. The states are very persistent, however switches occasionally occur. The lengthier the previous reassurance, the more likely the clinician is to stay in the current state.

**Conclusions:**

HMMs prove to be a valuable tool and provide important insights for practitioners.

**Trial registration:**

Trial Registration number: ClinicalTrials.gov: NCT02599506. Prospectively registered on 11th March 2015.

**Electronic supplementary material:**

The online version of this article (10.1186/s12874-018-0629-0) contains supplementary material, which is available to authorized users.

## Background

The experience of patients with cancer during their contact with health services has drawn increased attention by providers, the professions and quality management staff [1].The components of patient experience are hotly debated but a consensus opinion is easily found to support the view that good communication skills are a mandatory process to achieve high ratings of patient experience [2]. A key element of the clinician’s interactional abilities with their patients is to deliver reassurance to their patients [3]. Surprisingly the number of investigations that focus explicitly on reassurance giving by staff in clinical settings, let alone specifically in the cancer specialty, is low. Our group has identified a literature in reassurance giving applied to primary care consultations [4]. Where reference has been made to the cancer service setting the description and evidence base for the use of reassurance has tended to be cursory or very general in its recommendations. A recent review has focused on active surveillance of cancer patients and how this may function to provide reassurance [5]. However it does not focus on *how* reassurance can be given. Typically staff are encouraged to use reassurance but with little advice on when to use, the type of script that might be applied, and how frequently the strategy would be included in a consultation [6].

The definition of reassurance that has been advocated states that it is “verbal and non-verbal behaviours displayed by someone in an effort to reduce the concern of another” [7]. There are various types of reassurance and a helpful dichotomy is to divide reassuring statements commonly used by practitioners into two types, namely: cognitive and affective (see Appendix for more details). A clinician that adopted cognitive reassurance would provide informative statements, such as “these results are typical of what we find in patients who respond well to initial treatment intervention”. Affective reassurance, fits the convention of instructing the patient “not to worry”. A common response however from patients is that the exhortation from the clinician to switch off the mechanism of worrisome thoughts and feelings, is exactly when the patient believes they should worry. In other words the recommendation to stop worrying tends to do the reverse. Studies on the clinicians’ use of reassurances and patients’ immediate responses are virtually non-existent. Our group believes that this is an omission. Clinicians, we believe would welcome more basic information on the practice of this common strategy, commonly listed as a patient management competency to assist patients in accepting their diagnosis or treatment plan.

The work of our group has focused on the interaction between clinician and patient. We have expended effort and resource on developing coding of emotional expression in patients and the immediate response of their accompanying clinician [8]. This research has investigated the response of clinicians to emotional content of patient utterances. Inspection of various interactions in patients with cancer and their healthcare team members has shown the use of reassurance is quite frequent and varies throughout the consultation. A current data set that is pertinent to this careful inspection of reassurance giving is a data corpus of audio files of review consultations between patients with breast cancer and their therapeutic radiographer [9]. The review appointments are conducted weekly during the radiotherapy treatment that typically is daily apart from weekends and lasts one month duration. The purpose of the review meetings is to support the patient and answer questions about the course of treatment and possible side effects when they arise.

For each session a time series of reassurance type and duration as well as patient response type and duration were derived from the recording. With data already available, the challenge was to find an appropriate time series model for the clinicians’ reassurances. It is reasonable to assume that during a consultation clinicians go through different unobservable phases or states, in which they are more or less inclined to provide a particular type of reassurance. To put it more formally, the distribution of the reassurance type depends on the state and changes over the different states. Moreover, we would expect that the probability of the clinician being in a certain state is not independent of the past but rather depends on the state the clinician was in during the previous reassurance. This renders Hidden Markov Models (HMMs) suitable candidates for modeling time series of reassurance types. Among the advantages of HMMs are intuitive appeal, mathematical tractability and flexibility. Indeed, extension such as allowing covariates to influence the probability of switching between states or fixed effects for the different clinicians are straight forward to implement. HMMs are gaining popularity in medicine having been applied for example for modelling sleep disorders [10] as well as for monitoring circadian rhythmicity [11].

The global aim of this study is to learn about the nature of reassurances, produced by clinicians during sessions with breast cancer patients. Of particular interest is to identify key factors that influence the clinician’s behaviour.

## Methods

### Data

The data sets consists of 483 reassurances from the first session with 44 patients. Each session was held by one of the two available clinicians. Each reassurance was coded as a binary variable, taking the value 1 when Cognitive and 0 when Affective.

The coding scheme was drawn from [12]. Theoretically, reassurance is offered by clinicians when they sense the patient is anxious and can be expressed into two fundamental types. The clinician can respond to a patient concern by providing information (e.g. “the likelihood that your cancer will return is very rare”) and may indirectly reduce patient anxiety. Alternatively, the clinician response may be directly associated to the patient’s expression of anxiety by recognising the patient’s emotion and suggesting how to manage (e.g. “there is no need to worry”). Strict rules were detailed in a coding manual. When a turn was found to contain a “reassurance” the coder is required to code either as cognitive or affective. If there is doubt about the assignment, which very occasionally occurs due to a lack of words used by the radiographer to provide clarity between cognitive and affective (that is, too few to provide sufficient context), then the priority is to code “cognitive”.

The data set also contains a binary variable of the patient responses, taking the value 1 when the response is positive and 0 when it is neutral (See Appendix for more details of the coding scheme and examples). In addition, the duration (in seconds) of both reassurances and responses was noted together with the encoded ID of the clinician (taking the values 1 and 2).

In our analysis we consider time series of reassurances ordered by time of occurrence. The time between the reassurances is not of importance in the way we define the time series. Our approach is similar to a study of biological behaviour[13], where the authors model sequences of dives of blue whales. The full data consists of a series of short samples as shown in Table 1. Here by sample we mean the time series of reassurances in a single session. The sample sizes, i.e. the number of reassurances for a given session, can be as small as 3, while the largest sample size is 37.

**Table 1:** Frequencies of the sample sizes from the 44 sessions

Size	3	4	5	6	7	8	9	10	12	13	14	15	16	18	19	26	37
Freq.	3	4	2	1	3	3	5	4	4	6	1	1	1	1	3	1	1

**Fig. 1:**
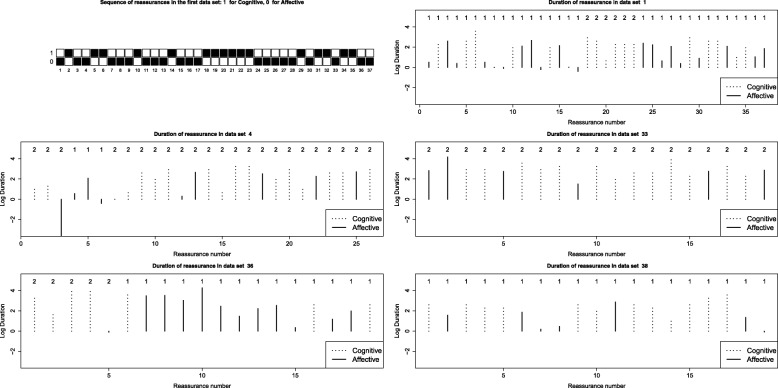
Pooled reassurances. Top-left corner - Ordered reassurances from the first session (1 = Cognitive, 0 = Affective). The filled boxes indicate which of the two types was used for a given reassurance. The remaining 5 plots show the duration and the type of reassurance in the five richest data sets. The numbers give the decoded state according to the 2-state HMM with log duration as a covariate

Figure 1 provides important insights on the data. The sequence of reassurances of the richest data set (session 1) is plotted in the top-left corner. A filled box indicates whether the respective reassurance was cognitive (upper row) or affective (lower row). It provides some indications that bouts of cognitive reassurances are followed by bouts of affective ones - a case for Hidden Markov Models. It also indicates that there is no strong preference for any of the two types. Indeed, in the pooled sample of all 483 observations 55.9% of the reassurances are cognitive and 44.1% are affective. The remaining five plots in Fig. 1 provide information on the log duration as well as the type of the reassurances in each of the five largest data sets. In terms of duration cognitive reassurances seem to last longer than the affective ones. This is to be expected as cognitive reassurances contain more information in the form of verbal content than the affective ones.

### Hidden Markov models

We fit Hidden Markov Models (HMMs) to the 44 data sets. An HMM is a mixture model consisting of two components: an observable time series and an underlying latent state sequence. The observable time series for a single data set, denoted by *X*_*t*_, *t*=1,…,*n*, in our case relates to the type of reassurance and is thus Bernoulli (or binary) taking the value 1 if the reassurance is cognitive and 0 otherwise. Each observation, i.e. each reassurance, is assumed to be the realization of one of *N* state-dependent distributions, binary in our case, each with probability *π*_*i*_, *i*=1,...,*N*, of “success” (in our case a cognitive reassurance). In our study we focus on a two-state model, i.e. we set *N*=2, but check in our empirical study whether an increase in the number of states is necessary.

**Fig. 2:**
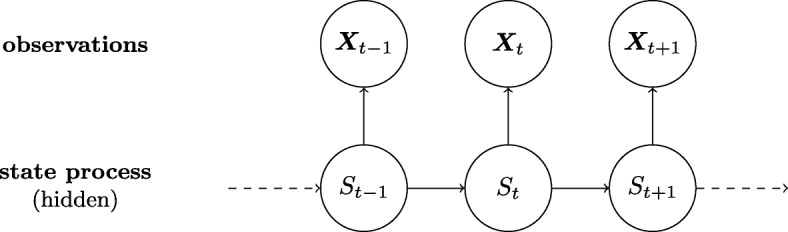
Visualisation of an HMM. Arrows indicate dependence. Here, the state process S is the “behavioural” state of the clinician, and the observations X are the types of reassurances, with subscripts indicating the ordered reassurances

The state process, denoted by *S*_*t*_, *t*=1,…,*n*, takes values in the set {1,...,*N*} and determines which of the *N* distributions the observation *X*_*t*_ is drawn from. The Markov property is assumed for the state process, so that *S*_*t*_ depends only on the previous state variable *S*_*t*−1_. Conditional on the current state *S*_*t*_, the observable variable *X*_*t*_ is independent of all past observations and states. The two components of an HMM with their dependence structure are visualised in Fig. 2.

#### Baseline model

The Markov Chain of the latent process is characterized by the initial distribution of the chain and the transition probabilities Pr(*S*_*t*_=*j*|*S*_*t*−1_=*i*), i.e. the probability of being in state *j* at time point *t* given that the process is in state *i* at time point *t*−1, for all *i*,*j* and *t*. For the baseline model [14], the state transition probabilities,

$$\gamma_{ij}:=\text{Pr}\left(S_{t}=j|S_{t-1}=i\right), $$

are assumed to be constant over the time, i.e. the Markov chain is homogeneous. We summarize these probabilities in a *N*×*N* transition probability matrix (t.p.m.) ***Γ***. When *N*=2 the t.p.m. is given as:

1 $$ \boldsymbol{\Gamma}= \left(\begin{array} {cc} \gamma_{11} & \gamma_{12}\\ \gamma_{21} & \gamma_{22} \end{array} \right) = \left (\begin{array} {cc} 1 - \gamma_{12} & \gamma_{12}\\ \gamma_{21} & 1 - \gamma_{21} \\ \end{array} \right)   $$

The two matrices are equivalent because the rows sum to unity. The initial state probabilities are summarized in a row vector ***δ***, where *δ*_*i*_=Pr(*S*_1_=*i*). When the Markov chain is homogeneous, we assume that the process is in equilibrium when we start observing it, such that the initial distribution is the stationary distribution. In this case ***δ*** is the left eigenvector of ***Γ***, associated with the eigenvalue 1. It can be easily calculated as the solution of the system of linear equations ***δ***(***I***_*N*_−***Γ***+***U***)=***1***^′^, where ***I***_*N*_ is an *N*×*N* identity matrix, ***U*** is an *N*×*N* matrix of ones and ***1*** is a conformable column vector of ones.

For the state-dependent distribution of *X*_*t*_ we assume the Bernoulli distribution, with probability of a cognitive reassurance varying across the states. More precisely for *N*=2 we assume that:

2 $$ X_{t}|S_{t}= i \sim \text{Bernoulli}(\pi_{i}), \quad i=1,2,   $$

so that

$$\begin{aligned} &\text{Pr}(\text{cognitive reassurance}|S_{t}=i) = 1 - \\ & \qquad \text{Pr}(\text{affective reassurance}|S_{t}=i) = \pi_{i}, \end{aligned} $$

with *π*_1_≠*π*_2_ in general.The likelihood can be expressed using the following matrix product:

3 $$ \mathcal{L}(\boldsymbol{\theta}| x_{1},...,x_{n}) = \boldsymbol{\delta} \boldsymbol{P}(x_{1}) \boldsymbol{\Gamma} \boldsymbol{P}(x_{2}) \times... \times \boldsymbol{\Gamma} \boldsymbol{P}(x_{n}) \boldsymbol{1},   $$

where ***θ*** denotes the vector with the parameters to estimate; ***P***(*x*_*t*_) is an 2×2 diagonal matrix with the conditional probability mass functions

$$ P\left(X_{t}=x_{t}|S_{t}=i\right) = \left\{ \begin{array}{ll} \pi_{i} & \text{for a cognitive reassurance} \\ 1- \pi_{i} & \text{for an affective reassurance} \end{array} \right. $$

of *X*_*t*_, given *S*_*t*_=*i*, *i*=1,2, on the main diagonal; ***1*** is as before a column vector of ones.

To illustrate how the likelihood function is constructed for a two-state HMM consider again the t.p.m. given in Eq. (1) and the state-dependent distributions in (2). When the Markov chain is in equilibrium at the start of the time series, then

$$\boldsymbol{\delta} = \frac{1}{\gamma_{12}+\gamma_{21}} \cdot (\gamma_{21},\gamma_{12}), $$

which is the stationary distribution. In the first data set the reassurances are (0,1, …,0) (where the dots indicate general observations which we do not detail here for reasons of space). The corresponding likelihood is:

$$\begin{array}{*{20}l} \mathcal{L}(\boldsymbol{\theta}| 0,1,...,0) \,=\, & \frac{1}{\gamma_{12}+\gamma_{21}} (\gamma_{21},\gamma_{12}) \left (\begin{array} {cc} 1- \pi_{1} & 0\\ 0 & 1- \pi_{2} \\ \end{array} \right) \\ \phantom{=} & \times\! \left (\begin{array} {cc} 1 - \gamma_{12} & \gamma_{12}\\ \gamma_{21} & 1 - \gamma_{21} \\ \end{array} \right) \left (\begin{array} {cc} \pi_{1} & 0\\ 0 & \pi_{2} \\ \end{array} \right)\; \ldots \; \\ \phantom{=} & \times\! \; \ldots\! \; \!\left (\! \begin{array} {cc} 1 - \gamma_{12} & \gamma_{12}\\ \gamma_{21} & 1 - \gamma_{21} \\ \end{array} \!\right)\! \!\left (\!\begin{array} {cc} 1- \pi_{1} & 0\\ 0 & 1- \pi_{2} \\ \end{array}\! \right)\! \left(\begin{array}{c} 1 \\ 1 \end{array} \right), \end{array} $$

where ***θ***=(*γ*_12_,*γ*_21_,*π*_1_,*π*_2_)^′^. To fit an HMM to our data, we assume that the 44 samples are independent and that the model parameters are identical across all sessions. The independence assumption is reasonable - each session is with a different patient and is spaced in time. The second assumption will be relaxed when we consider different effects for the two clinicians. Under these assumptions, the joint log likelihood is simply the sum of the 44 individual log likelihoods, obtained from the likelihood in (3). The joint log likelihood is numerically maximised using a Newton-Rapson-type optimisation procedure. We implement the numerical maximisation using the routine nlm() in the statistical software R [15].

#### HMMs with covariates

When considering covariates in an HMM, it is reasonable to assume that the external variables directly influence the transition of states rather than the state-dependent distributions, which remain fixed for a given state. This allows us to draw conclusions about the effect of, for example, a positive patient response on the transitions between the preferences of the clinicians for different types of reassurances.

Let $\boldsymbol {z}^{\prime }_{t} = (1,z_{1,t},...,z_{k,t})$ be a vector of *k* covariates at time *t*. Then for a series of covariate vectors, ***z***_1_,***z***_2_,***z***_3_,..., the model given in the previous subsection was modified by letting the transition probabilities depend on the covariate values as follows:

4 $$\begin{array}{*{20}l} \gamma^{t}_{12} & = \text{Pr}\left(S_{t}=2|S_{t-1}=1\right) \\&= \text{logit}^{-1}\left(\beta_{1,0} + \beta_{1,1} z_{1,t} + \ldots + \beta_{1,k} z_{k,t}\right) =  \\ & = \text{logit}^{-1}\left(\boldsymbol{\beta}_{1}^{\prime}\boldsymbol{z}_{t}\right)  \end{array} $$

5 $$\begin{array}{*{20}l} \gamma^{t}_{21} & = \text{Pr}\left(S_{t}=1|S_{t-1}=2\right) \\&= \text{logit}^{-1}\left(\beta_{2,0} + \beta_{2,1} z_{2,t} + \ldots + \beta_{2,k} z_{k,t}\right) =  \\ & = \text{logit}^{-1}\left(\boldsymbol{\beta}_{2}^{\prime}\boldsymbol{z}_{t}\right),  \end{array} $$

where logit^−1^ is the inverse of the multinomial logistic link function and the vectors $\boldsymbol {\beta }^{\prime }_{1} = \left (\beta _{1,0}, \beta _{1,1}, \ldots, \beta _{1,k}\right) $ and $\boldsymbol {\beta }^{\prime }_{2} = \left (\beta _{2,0}, \beta _{2,1}, \ldots, \beta _{2,k}\right) $ contain the 2*k*+2 coefficients in the t.p.m. to be estimated. The baseline model is a special case, in which all beta coefficients except for *β*_1,0_ and *β*_2,0_ are zero.

The elements on the main diagonal of the t.p.m. $\left (\gamma ^{t}_{11}\right.$ and $\left.\gamma ^{t}_{22}\right)$ are obtained by subtracting the other entry on the same row from one. Note that the superscript *t* indicates that the transition probabilities are no longer constant but vary over time. Thus the Markov Chain is no longer homogeneous and we cannot assume that the state process is stationary. We address this issue by treating the parameters of the initial distribution *δ*_1_ and *δ*_2_=1−*δ*_1_ as additional model parameters and add *δ*_1_ to the vector ***θ*** of parameters to estimate. The formulation of the state-dependent distribution remains otherwise unchanged as is the construction of the likelihood.

#### HMMs with fixed effects for the clinicians

The 44 first sessions were held by two different clinicians in a major regional cancer centre in Scotland. The therapeutic radiographer consisted of an experienced staff member with some managerial responsibility and a counselling qualification, whereas the second staff member was recently qualified. Each session is held by only one of the two clinicians, labeled 1 and 2. It is natural to assume that the nature of the latent processes for the two clinicians, in particular the transition probabilities between the two states, differs. We account for this modification by allowing fixed effects in the t.p.m. For the baseline model this means that for a given session ***Γ*** in Eq. (3) equals one of the two matrices

6 $$ \boldsymbol{\Gamma^{1}}\,=\, \left (\begin{array} {cc} 1 - \gamma^{1}_{12} & \gamma^{1}_{12}\\ \gamma^{1}_{21} & 1 - \gamma^{1}_{21} \\ \end{array} \right) \quad \text{or} \quad \boldsymbol{\Gamma^{2}}\,=\, \left (\begin{array} {cc} 1 - \gamma^{2}_{12} & \gamma^{2}_{12}\\ \gamma^{2}_{21} & 1 - \gamma^{2}_{21} \\ \end{array} \right)   $$

The superscript in this case denotes the clinician ID. When the first clinician is holding the session ***Γ***=***Γ***^***1***^, while ***Γ***=***Γ***^***2***^ when the second clinician is working. Therefore the ID of the clinician determines which of the two t.p.m.s is “switched on” for the session.

The modification in the case of covariates in the t.p.m. is straightforward. The t.p.m.s in Eq. (3) are calculated using the following equations

$$\begin{array}{*{20}l} \text{Clinician 1:} \qquad \gamma^{t,1}_{12} & = \text{logit}^{-1}\left(\boldsymbol{z}^{\prime}_{t}\boldsymbol{\beta}^{1}_{1}\right) \qquad \\ \gamma^{t,1}_{21} & = \text{logit}^{-1}\left(\boldsymbol{z}^{\prime}_{t}\boldsymbol{\beta}^{1}_{2}\right), \end{array} $$

$$\begin{array}{*{20}l} \text{Clinician 2:} \qquad \gamma^{t,2}_{12} = \text{logit}^{-1}\left(\boldsymbol{z}^{\prime}_{t}\boldsymbol{\beta}^{2}_{1}\right)\\ \phantom{\text{Clinician 2:}} \qquad \gamma^{t,2}_{21} = \text{logit}^{-1}\left(\boldsymbol{z}^{\prime}_{t}\boldsymbol{\beta}^{2}_{2}\right) \end{array} $$

The initial distributions $\boldsymbol {\delta ^{1}} =\left (\delta ^{1}_{1},1-\delta ^{1}_{1}\right)$ and $\boldsymbol {\delta ^{2}} = \left (\delta ^{2}_{1}, 1- \delta ^{2}_{1}\right)$, where the superscript indicates the ID of the clinician, are estimated separately.

#### Model selection

Since we are considering several candidate models, we need formal model selection criteria. We choose to work with the widely used Akaike Information Criterion (AIC), which is given as

$$\begin{array}{*{20}l} \text{AIC} &= -2 \log \ell + 2p \\ \end{array} $$

where log*ℓ* is the joint log-likelihood for the 44 data sets, *p* is the number of parameters estimated, i.e. the length of the vector ***θ***. Among the candidates we choose the model with the lowest AIC.

#### Global decoding

The states are not observable but a procedure known as the Viterbi algorithm (see [14]) allows us to obtain the *sequence* of states with the highest probability given the data. In particular, it seeks the sequence *s*_1_,*s*_2_,...,*s*_*n*_ that maximizes the conditional probability:

$$\text{Pr}(S_{1} = s_{1},..., S_{n} =s_{n}| X_{1}=x_{1},...,X_{n} = x_{n}) $$

or, equivalently:

$$\text{Pr}(S_{1} = s_{1},..., S_{n}= s_{n}, X_{1}=x_{1},...,X_{n} = x_{n}) $$

Viterbi is a recursive algorithm for solving this optimization problem.

## Results

We conduct an empirical study, in which we fit HMM of different complexity to the time series of reassurances. In a first step we justify the use of a 2-state HMM as well as the need to include a dependence structure in the latent process. For this purpose we fit a baseline model with 1, 2 and 3 states. An HMM with 1 state is a trivial case of independent observations. We also fit an independent mixture model, which is a special case of an HMM, in which the states are independent of the past (the visualisation is similar to that of a classical HMM in Fig. 2 with the arrows between states *S*_*t*_ and *S*_*t*+1_ removed). Table 2 provides the AIC and the negative log-likelihood of the four fitted models.

**Table 2:** Comparison of the model selection criteria for 4 baseline models and 6 models with covariates. FE stands for Fixed effects, DOPR for duration of previous response, and nllk stand for negative log likelihood

	nllk	AIC
Baseline *N*=1	328.95	659.90
Baseline *N*=2	320.69	649.38
Baseline *N*=3	317.53	653.05
Indep. mixt.	328.95	665.90
Response Type	318.30	650.59
Log DOPR	316.63	**647.25**
Response Type & log DOPR	315.71	649.42
FE baseline	316.04	648.09
FE Response	314.56	653.04
FE Log DOPR	313.66	651.32

The lowest AIC of all models is given in bold

We find some evidence for the use of a hidden state structure as the baseline 2-state HMM has the lowest AIC. The 3-state HMM does not bring an improvement so 3 states seem to be too much in this context. The independent mixture model provided the worst fit and we therefore conclude that there is dependence in the observed data.

Before we proceed with the models with covariates, we first consider the estimates from the baseline 2-state HMM given below:

$$\begin{array}{*{20}l} \hat \pi_{1} & = 0.209, \qquad \hat \pi_{2} = 0.782 \\ \hat \gamma_{11} & = 0.735, \qquad \hat \gamma_{21} = 0.169,\\ \qquad \hat \gamma_{12} &= 0.265, \qquad \hat \gamma_{22} = 0.831 \end{array} $$

Based on the estimates of the “success” probabilities we can interpret state 1 as the dominantly affective state and state 2 as the dominantly cognitive state. Note that cognitive reassurances can still occur in state 1 and, similarly, affective reassurances can still occur in state 2. This just happens with a low probability (around 20% for both cases). The states are persistent - the probabilities of remaining in the same state are relatively high (0.735 for state 1 and 0.831 for state 2).

In a next step we fit 2 models with one covariate in the t.p.m. Our first choice for a covariate is the patient’s response. We argue that the previous rather than the current response is more likely to influence the transition of states and therefore use lagged values. This occurs at no cost as we have an observation of the lagged covariate for each transition. The initial distribution is not affected by covariates and is estimated separately. The second covariate we use is the log duration of the previous reassurance (log DOPR), which is obviously also lagged. We use log duration rather than duration, motivated by the fact that duration is heavily skewed with a few large outliers. We also fitted a model with two covariates - response type and log DOPR - to check whether adding more variables will improve the fit. Finally we fit all three models (baseline and two different covariates) allowing for fixed effects for the two different clinicians as discussed in the previous section. The negative log likelihood and the AIC of the 6 additional models are given in Table 2.

The model with covariates that brings the largest improvement over the baseline HMM is the model with log DOPR. The length of the previous reassurance seems to have an effect on the transition probabilities between the states. We will discuss this model in more detail in the subsection below. Interestingly, the HMM with Response Type is not preferred over the baseline model. Compressing the patient’s response into a binary variable (“neutral”, “positive”) might have lead to some information loss. The inclusion of two covariates is not justified as the AIC of the model with log DOPR and Response Type is higher than the one of the model with log DOPR only.

In the absence of other covariates, it seems to matter which of the two clinicians is holding the session. However, adding fixed effects to the models with covariates in the t.p.m. does not bring a significant improvement over the simpler models. A logical conclusion in this case is that clinicians react similarly to patient responses or, respectively, to their own lagged reassurance. In summary, from the 10 competing models, the one with lowest AIC is the 2-state HMM with log DOPR as a covariate and thus it is our preferred model. We investigate it below.

### 2-state HMM with log DOPR

The estimates from the model fit together with the respective 95% confidence intervals (lower and upper bounds) obtained from the inverse Hessian are:

**Table:** 

Parameter	Estimate	Lower bound	Upper bound
*π*_1_	0.408	0.307	0.519
*π*_2_	0.797	0.620	0.905
*β*_1,0_	-64.727	-459.637	330.182
*β*_1,1_	-185.917	-1291.193	919.359
*β*_2,0_	-1.014	-2.735	0.709
*β*_2,1_	-0.423	-1.209	0.349
*δ*_1_	0.363	0.117	0.711
*δ*_2_	0.637	0.289	0.883

The estimated parameters of the state-dependent distributions (*π*_1_ and *π*_2_) are in line with those of the baseline model. Therefore we retain the labels given to the states in the previous section. The probability of starting the session in the dominantly cognitive state is estimated as being almost twice as large the respective probability of starting the session in the dominantly affective state.

The interpretation of the *β* coefficients is not straightforward. To gain insight of the influence of the log DOPR on the latent process, we examine the transition probabilities and the stationary distribution for given values of the covariate (see [16]). Assuming that log DOPR is fixed at a certain level for all observations, we treat the HMM as homogeneous and stationary, i.e. it becomes the baseline model. Then we can calculate the hypothetical transition probabilities *γ*_12_ and *γ*_21_ from (4) and (5), respectively. Varying the value at which we fix log DOPR allows us to present *γ*_12_ and *γ*_21_ as functions of the log DOPR.

**Fig. 3:**
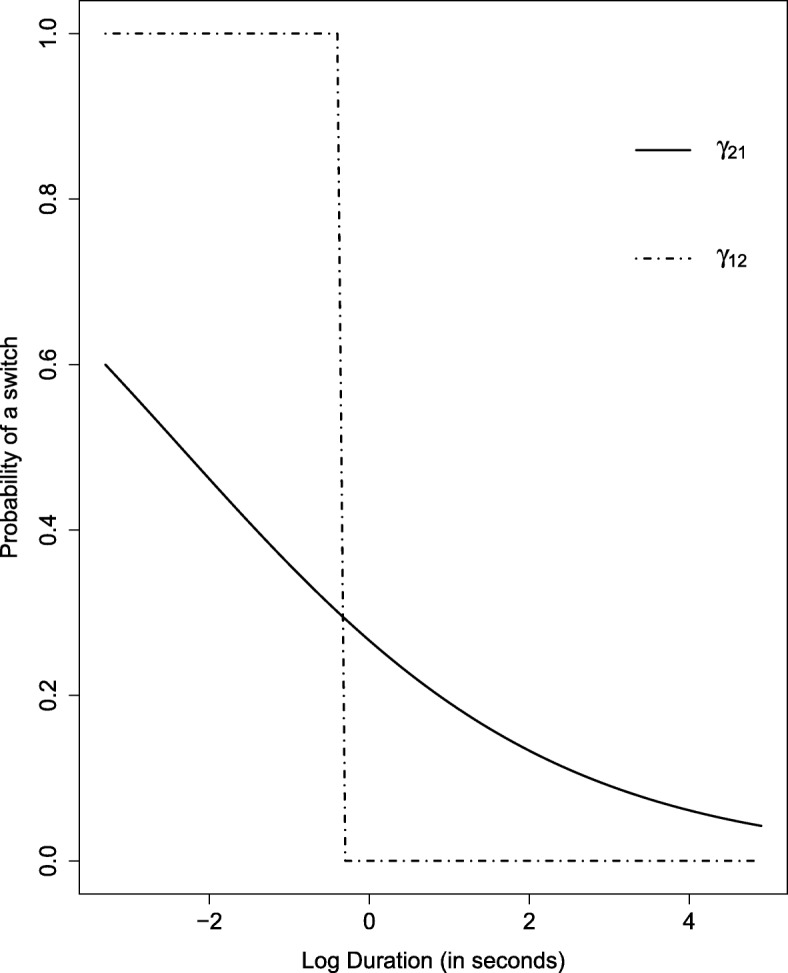
Transition probabilities as function of log DOPR. Dashed line gives *γ*_12_ as a function of log DOPR, while the solid line gives *γ*_21_ as a function of log DOPR

For this part of the analysis we treat the estimated parameters in the ***β*** matrix as the true ones. The results are plotted in Fig. 3. Both probabilities indicate that as the DOPR increases, the probability of switching into a different state decreases. In that sense when the previous reassurance has taken a reasonable amount of time, i.e. more than just a few seconds, the states become very persistent - a finding that is in line with our analysis of the baseline model. The behaviour of the transition probability from state 1 to state 2 is quite abrupt, which can be attributed to the large estimates of *β*_1,0_ and *β*_1,1_.

We advise caution when interpreting the influence of log DOPR. The importance of this covariate, indicated by the improvement in the AIC over the baseline model, is not confirmed by the confidence intervals of *β*_1,1_ and *β*_2,1_, which cover the zero.

We explain the large uncertainty around the estimated parameters with the small sample size and the fact that under the model there are few transitions. Next we look at the results from the global decoding applied on the five richest data sets (Fig. 1). The states are indeed quite persistent - the clinicians spend a lot of time in the same state but occasionally switches occur, especially after a short reassurance. Some sessions, however, are entirely spent in the same state.

## Discussion

The use of reassurance in clinical communication has been poorly studied in health care communication research. Whereas there is a recognition of the importance of the clinician behaviour in response to a concern expressed by the patient as evidenced by inclusion in behavioural coding schemes of Doctor:Patient consultations such the RIAS [17] and the VR-CoDES [8] systems, there are few examples of in-depth description of the behaviour. The formulation of dividing reassurance into two discrete types labelled cognitive and affective has led to investigation of the frequency of each of these types in different conditions, patterns within consultations and the influence of each type on patient’s psychological, behavioural and health outcomes [4]. Our study has focused on a detailed description of frequency and patterning. Attempts to understand the effect of these reassurance patterns on outcome are outside the scope of our investigation.

From a descriptive viewpoint, it is clear that reassurance giving is repeated many times over the course of these review consultations of the patient with their therapeutic radiographer. Previous work is limited on the effect of the radiographer on their patient. An early report has stated that the communication of the radiographer has an important part to play in the experience of pain felt by the patient with breast cancer [18]. This research report was limited to the mammography assessment only and did not incorporate direct observations. The evaluation relied on questionnaire ratings given by the patient only. Our study aimed at uncovering probabilistic patterns of reassurance giving, allowing for the clinicians to go through different, hidden behavioural states. This could be elegantly done using the HMM approach.

HMMs have proved to be a useful tool in various fields of science - finance, computational linguistics, statistical ecology, meteorology among others [19–22]. In this paper we have demonstrated that health care communication and health psychology make no exception. In fact [23] suggest that HMM could be used to model “the complex structure of doctor patient consultation” but found no applications in this field. In this paper we aimed to fill in this gap.

Our analysis found indications for the existence of two states in the clinicians’ behaviour. During sessions the radiographers occasionally undergo changes in their inclination to give cognitive and, respectively, affective reassurances. Note that the states in a Hidden Markov Model are a mathematical construct, which may or may not have a straightforward interpretation in the context of a particular study. We cautiously labelled the states “dominantly affective” and “dominantly cognitive”. Our interpretation is that in a “dominantly affective” (“dominantly cognitive”) state the clinician is more inclined to give an affective (cognitive) reasurance. However, we insist that we do not interpret the states as mental states or moods.

We explored the influence of covariates such as type of patience response and (log) duration on the probabilities of switches between the states, while at the same time accounting for the difference between the two clinicians in the study (fixed effects). The model discrimination criterion used in this study, the AIC, preferred the model with the log duration. Including fixed effects and adding (further) covariates brought improvements in the likelihood, which however did not justify the increased number of parameters.

An important insight from our best model is that for reasonably long previous reassurances the states become very persistent. The baseline model without covariates also indicated persistency of the states. We can conclude that although clinicians occasionally change their approach, there is not much flexibility. Data was one of the main limitations of our approach. A collection of very short data sets pose a challenge for time series models such as HMMs. In particular, when the switches are not many, the uncertainty around the estimated parameters of the covariates is large. Longer time series, if possible, would provide clearer answers as to the importance of the covariates and would potentially strengthen the argument for the existence of hidden states.

When fitting HMMs we assumed independence of the different sessions. We argue that this is a justified assumption because for each session the clinicians face a different patient and have had time to “reset” from the previous patient. Moreover, only 40% of patients going to the cancer centre were recorded - this makes 2 patients on average in a 2-h consultation slot (5 patients per slot). The chances of having back-to-back recorded sessions are therefore quite low. Moreover, we want to emphasize that the professionalism of the staff is excellent and even with daily variation in personal motivation effects such as tiredness and low mood would be very hard to identify in their clinical manner.

The importance of this work clinically is to concentrate attention of cancer care researchers on the importance of sequencing of certain communication skills techniques applied in routine delivery of treatment. Previous work has concentrated on evaluating skills programme interventions through simple patient ratings and less on direct observables [24]. A recent literature review has focused on the psycho-social support offered by health professionals during radiation treatment who have direct daily contact with patients [25]. It recommended explicit questioning by staff to elicit distress in patients especially during the early sessions of treatment. One study has designed specific workshop interventions for improvement of radiation staff communication skills. One of the components was the identification of emotional cues and responding appropriately. An important skill taught in these workshops (amongst other skills) was the avoidance of simple reassurance [26]. The influence of this approach still awaits evaluation and testing of reduced patient distress levels. Hence future work should concentrate on the observation of the technique of reassurance, the specific type utilised, its sequence and ability to reduce distress in patients undergoing, what is for many patients with breast cancer, the last major primary curative treatment prior to discharge and follow-up.

## Conclusion

Our work is the first attempt to model the behaviour of a small set of clinicians with patients undergoing radiotherapy who express anxieties during their review appointments with their therapeutic radiographer. The important feature of our results is twofold: first that we have been able to apply a powerful statistical model to explain with some success reassurance behaviour in routine appointments in a major cancer centre. Second we have found that the type of reassurance behaviour (cognitive or affective) is not randomly exhibited but demonstrates a pattern that may have implications for patient psychological outcomes. Although somewhat preliminary we are enthusiastic about sharing this with the oncology community which may regard reassurances as a straightforward clinical skill.

## Additional file

Additional file 1 Coding rules and reassurance coding scheme with examples. (DOCX 23kb)

## Abbreviations

AIC: Akaike Information Criterion
FE: Fixed effects
HMM: Hidden Markov Model
DOPR: Duration of the previous reassurance
