# Supplementary material for: Modelling reassurances of clinicians with hidden Markov models
Source: BMC Med Res Methodol. 2019 Jan 9;19:11. doi: 10.1186/s12874-018-0629-0 (PMC6327545; doi:10.1186/s12874-018-0629-0)
Supplement: Supplementary file 1 — Coding rules and reassurance coding scheme with examples. (DOCX 23kb) [file 12874_2018_629_MOESM1_ESM.docx]

Appendix

Reassurance was coded into affective and cognitive elements. The scheme was based on previous work by Holt and Pincus (2016). Only “explicit”, verbal forms of reassurance were coded. It is acknowledged that non-verbal expressions of the face or postural movements, say with the hands, for example, can signal reassurance.

Reassurance was coded if it arose in response to a concern, question or emotional cue. Affective reassurance referred to expressing empathy, encouragement and generic, emotional reassurance. Cognitive reassurance referred to the provision of information, advice and explanations regarding any element of treatment, cancer, future care and prevention. Full definitions and examples of these terms are presented below.

Coding was competed using a turn style basis: that is, one stream of speech was one “turn”. A new turn began when the speaker changed, or when 5 seconds had elapsed with no speech by anyone. In this instance, the end time for the turn was following the last word spoken. Only one code was assigned per turn.

Patient response was coded as positive or neutral, based on the interpretation by the coder (AB) under supervision of the principal investigator of the project sited in the regional cancer centre, namely GH. The patient was given up to 5 seconds to respond to the reassuring statement. If there was no response within this time frame, the response was coded as neutral. A positive response was one that was irrefutably positive in nature, dependent on speech content and tone. Responses that were considered equivocal from detailed inspection by coder and supervisor were coded as neutral. In addition, instances where patient questions or patient silences were responses to reassuring statement of radiographer were coded as neutral responses when given following a reassuring statement.

Intra-rater reliability was checked using the Kappa statistic. 10% of coded videos were recoded four weeks following initial coding. The coding demonstrated high reliability (0.78).

The following behaviours were taken from the cognitive and affective reassurance maps from Holt and Pincus (2016) to form the scheme:

- Information (see criteria in coding table)
- Generic reassurance (see criteria in coding table)
- Empathy/acknowledgement of concerns
- Encouragement/Empowerment (combined to form one code)

Following a trial run of this scheme, 2 new codes were added (see criteria in coding table):

- Further tests
- Advice giving

Advice giving has previously been identified as a form of cognitive reassurance by Pincus et al (2013), and was deemed suitable for this population.

**Reassurance coding scheme with examples**

Style Codes Examples

Affective Acknowledgment and Empathy “I can totally understand that”

*Statements clearly indicating that the* “you just want it done and dealt with

*provider is aware and accepting of the* as quickly as possible”

*concerns/issues of the patient;* “It’s a long road isn’t it, I know”

*statements portraying empathy*

Generic Reassurance “but you’re nearly there”

*General* *statements made to emotionally* “you managed pretty well last time

*reassure (simple and complex)* around, we’ve got no reason to

believe you won’t manage this time”

Encouragement/Empowerment “perfect. good. Well you’re doing

*Encouraging patient actions and* everything right”

*provide patient with a sense of control* “well you know better than anyone

else what you feel up to doing”

Uncertainty is not an issue “I don’t know the stats on that myself

*Statements that acknowledge uncertainty* ...but they always drum into us...

*But detract from its negativity* so it’s got to have some effect”

Cognitive Information/Explanations “that’s just because they’ve taken

*Provision of information or explanations* some tissue from the breast”

*Regarding any element of the treatment* “you’ always get these twinges and

*or cancer* aches and pains after any operation,

and they can last up to two years”

Advice “if you really wanted to go (swim)

*Suggestions given regarding behaviour*  …maybe say keep it to once a week”

Further tests “I think it’s worthwhile getting you

*Providing negative results/organising*  seen by one of the doctors, just

*follow up tests* to sound your chest”

Holt, N. & Pincus, T. (2016). Developing and testing a measure of consultation-based reassurance for people with low back pain in primary care: a cross-sectional study. *BMC Musculoskeletal Disorders, 17*(277). doi:10.1186/s12891-016-1144-2

Pincus, T., Holt, N., Vogel, S., Underwood, M., Savage, R., Walsh, D.A. & Taylor, S.J.C. (2013). Cognitive and affective reassurance and patient outcomes in primary care: A systematic review. *Pain, 154*(11), 2407-2416. doi:[10.1016/j.pain.2013.07.019](https://doi.org/10.1016/j.pain.2013.07.019)
